# Exosomal miRNA-profiling of pleural effusion in lung adenocarcinoma and tuberculosis

**DOI:** 10.3389/fsurg.2022.1050242

**Published:** 2023-01-06

**Authors:** Xuede Zhang, Lingling Bao, Guohua Yu, Haifeng Wang

**Affiliations:** ^1^ Department of Oncology, Weifang People's Hospital, Weifang, China; ^2^ Department of Hematology and Oncology, Beilun District People's Hospital, Ningbo, China

**Keywords:** exosomes, miRNAs, pleural effusions, bioinformatic analysis, lung adenocarcinoma

## Abstract

**Background:**

Pleural effusion (PE) caused by lung cancer is prevalent, and it is difficult to differentiate it from PE caused by tuberculosis. Exosome-based liquid biopsy offers a non-invasive technique to diagnose benign and malignant PE. Exosomal miRNAs are potential diagnostic markers and play an essential role in signal transduction and biological processes in tumor development. We hypothesized that exosomal miRNA expression profiles in PE would contribute to identifying its diagnostic markers and elucidating the molecular basis of PE formation in lung cancer.

**Methods:**

The exosomes from PE caused by lung adenocarcinoma (LUAD) and pulmonary tuberculosis were isolated and verified by transmission electron microscopy. The exosomal miRNA profiles were identified using deep sequencing and validated with quantitative real-time PCR (qRT-PCR). We performed bioinformatic analysis for differentially expressed miRNAs to explore how exosomal miRNAs regulate pleural effusion.

**Results:**

We identified 99 upregulated and 91 downregulated miRNAs in malignant pleural effusion (MPE) compared to tuberculous pleural effusion (TPE). Seven differentially expressed miRNAs (DEmiRNAs) were validated by qRT-PCR, out of which 5 (71.4%) were confirmed through sequencing. Gene Ontology (GO) analysis revealed that most exosomal miRNAs target genes were involved in regulating cellular processes and nitrogen compound metabolism. According to the Kyoto Encyclopedia of Genes and Genomes (KEGG) pathway enrichment analysis, the exosomal miRNAs target genes were mainly involved in Fc gamma R-mediated phagocytosis, Rap1 signaling pathway, and breast cancer. The hub genes, including ITGAM, FOXO1, MAPK14, YWHAB, GRIN1, and PRF1, were screened through plug-in cytoHubba. The PFR1 was identified as a critical gene in MPE formation using single-cell sequencing analysis. Additionally, we hypothesized that tumor cells affected natural killer cells and promoted the generation of PE in LUAD *via* the exosomal hsa-miR-3120-5p-PRF1 axis.

**Conclusions:**

We identified exosomal miRNA profiles in LUAD-MPE and TPE, which may help in the differential diagnosis of MPE and TPE. Bioinformatic analysis revealed that these miRNAs might affect PE generation through tumor immune response in LUAD. Our results provided a new theoretical basis for understanding the function of exosomal miRNAs in LUAD-MPE.

## Background

Pleural effusion (PE), the accumulation of fluid in the pleural space, is the presenting symptom of numerous diseases. Lung cancer and pulmonary tuberculosis are the leading causes of PE. However, a clinical challenge is a differential diagnosis of tuberculosis pleural effusion (TPE) and lung cancer-related malignant pleural effusion (MPE). The diagnosis of MPE is always based on PE cytological analysis. However, the sensitivity of cytology is low (1), and finding effective prognostic biomarkers is a research hotspot. Pleural biopsy is an invasive procedure with a potentially high risk that may not provide a comprehensive biological profile of malignant tissue due to tumor heterogeneity. Liquid biopsy is a non-invasive, rapid, and safe alternative to tissue biopsy for cancer diagnosis, and it is likely to provide a thorough understanding of tumor heterogeneity (2–4).

Exosome-based liquid biopsies have been proposed for use in lung cancer diagnosis, prognosis, and surveillance in numerous studies in the past (5–7). Exosomes are small extracellular vesicles released by various cells into their microenvironment and are present in most body fluids (8, 9). They contain ncRNA, DNA, proteins, and lipids that derive from the parental cells, reflecting the characteristics of the donor cell (10). Exosomes are enriched in PE and may be used as biomarkers for malignant pleural mesothelioma (11), lung cancer (12), and tuberculosis (13).

Exosomes can also serve as a critical mediator of intercellular communication, transmitting biological signals like proteins, mRNAs, miRNAs, and DNAs to recipient cells, resulting in morphological and functional changes in the corresponding cells. The miRNAs are a family of small, non-coding, endogenous molecules involved in the post-transcriptional regulation of gene expression (14). Recent studies suggested that miRNAs are abundant in exosomes and are resistant to RNase degradation, making them useful for diagnosis, prognosis, treatment monitoring, and exploration of disease mechanisms (15–17). PE contains many immune cells, such as dendritic cells, macrophages, neutrophils, and T cells (18, 19). It is well known that angiogenesis and increased vascular permeability are essential for forming PE. Recently, numerous studies have suggested that exosomal miRNAs can affect mRNA expression/translation in the recipient cells, including various immune cells and endothelial cells, indicating that miRNAs may play potential roles in mediating intercellular communication, immunoregulation, and angiogenesis in tumor progression (20–22). Tumor-derived exosomes may play important roles in PE formation. However, the underlying mechanisms are unclear.

In this study, exosomes derived from PE of patients with lung cancer and pulmonary tuberculosis were isolated, and the miRNA profiles were analyzed to identify those which are differentially expressed. We unveiled the most important miRNAs for diagnosing lung cancer and characterized the biological progression of LUAD-induced PE. The study established the viability of exosome-associated miRNA signatures in PE as tumor biomarkers and explored the potential mechanisms underlying PE formation in LUAD.

## Materials and methods

### Patients and pleural fluid collection

PE samples from six LUAD and six tuberculosis patients were collected between July 2019 and December 2020 at the Beilun Branch Hospital of the First Affiliated Hospital of the Medical School of Zhejiang University. The PE fluid was collected and stored at −80 °C. LUAD was diagnosed by pathology or cytology. The patients with tuberculous PE were confirmed by bacteriology or clinical diagnosis, along with observing several distinguishing characteristics, including a high lymphocyte ratio and adenosine deaminase levels > 40 U/L. This study was approved by the regional ethical committee, approval No. 2019-53 (K), date of approval: 2019-04-17. Informed consent was provided by all participants.

### Isolation of exosomes

Exosomes were isolated from PEs using the ultracentrifugation technique described previously (23). Briefly, PE samples were centrifuged at 500 × g for 5 min to eliminate cells. The supernatant was centrifuged at 2000 × g for 10 min to remove the component from the samples. The PE supernatants were then centrifuged sequentially at 10,000 × g for 30 min and were filtered through a 0.22 µm membrane filter (Merck Millipore). It was then centrifuged at 100,000 × g for 2  h. The exosome pellet was washed once with PBS and centrifuged again. Finally, the pellets were resuspended in PBS and stored at 80 °C until further use.

### Transmission electron microscopy (TEM) analysis

The exosomes were resuspended in 50–100 µl of 2% PFA, and 5 µl of the exosomal suspension was added to a formvar-carbon-coated copper grid and washed with PBS. The formvar membranes were kept moist at every step, but the other side remained dry. The copper mesh was placed on 50 µl of 1% glutaraldehyde droplets for 5 min before being washed eight times in 100 µl of ultrapure water for 5 min. The copper mesh-bearing exosomes were incubated for 5 min with 50 µl of uranyl oxalate droplets (pH 7) and 10 min with 50 µl of methylcellulose droplets. The excess samples were blotted off the filter paper, and the grid was air-dried for 5–10 min. Exosome morphologies were observed using the FEI Tecnai G2 Spirit transmission electron microscope.

### Extraction of exosomal miRNA

Total RNA, including miRNA, was extracted from exosome samples using the miRNeasy MiniKit (Qiagen), according to the manufacturer's instructions. Total RNA was eluted with 30 µl of nuclease-free water (Ambion) and stored at −80 °C until further processing. RNA samples were qualified and quantified by agarose gel electrophoresis and NanoDrop ND-1000 (NanoDrop, USA) before deep sequencing analysis or qRT-PCR validation.

### Small RNA library preparation and sequencing

RNA samples were qualified and quantified using agarose gel electrophoresis and Nanodrop ND-1000 (NanoDrop, USA). Small RNA libraries were constructed using the NEBNext Multiplex Small RNA Library Prep Set for Illumina (NEB, Ipswich, MA, USA). Their quality was evaluated using Agilent Bioanalyzer 2100 (Agilent Technologies, USA). The libraries were denatured with 0.1 M NaOH to generate single-stranded DNA. Sequencing was performed for 50 cycles on the Illumina NextSeq 500 system following the manufacturer's instructions. FastQC examined sequencing quality.

### Validation of sequencing data by qRT-PCR

To confirm miRNA sequencing results, qRT-PCR was used to compare expression levels of randomly selected seven miRNAs between the two groups. For miRNA detection, the RNA was reverse-transcribed into cDNA before PCR. The amplification conditions were as follows: 95 °C for 10 min, followed by 40 cycles of 95 °C for 10 s and 60 °C for 60 s. The miRNA level was quantitatively determined using a real-time RT-PCR kit. has-miR935p was used as a reference as its expression level remains constant across different samples.

### Bioinformatic analysis

miRDeep2 was used to obtain the miRNA counts, which were normalized using counts per million (CPM). Significant differences among groups were assessed using edgeR analysis. The differential genes were identified using the criteria |log2FoldChange| ≥ 1.5 and *P* value ≤ 0.05. The target genes of the top ten upregulated and downregulated differentially expressed miRNAs (DEmiRNAs) were predicted by the intersection of miRDB (24) and Targetscan (25) databases. Gene Ontology (GO) annotation and Kyoto Encyclopedia of Genes and Genomes (KEGG) pathway enrichment analysis were performed for the target genes of DEmiRNAs. The Search Tool for the Retrieval of Interacting Genes (STRING; http://string.embl.de/) database was used to construct a protein-protein interaction (PPI) of target genes for DEmiRNAs with an interaction score > 0.7 (26). The PPI network was visualized using Cytoscape, and the cytoHubba plug-in was used to identify hub genes (27). Based on the degree of the genes in the PPI network, the top 20 ranked genes were identified as hub genes. Finally, single-cell RNA profiles of MPE samples of GSE185058 were obtained from GEO (http://www.ncbi.nlm.nih.gov/geo), and differential genes were screened by iDEP.96 (http://bioinformatics.sdstate.edu/idep/) (28). The intersection of miRNA target genes and differential genes was expected to play a vital role in forming PE.

## Results

### Characterization of exosomes from Pe

**Figure 1:**
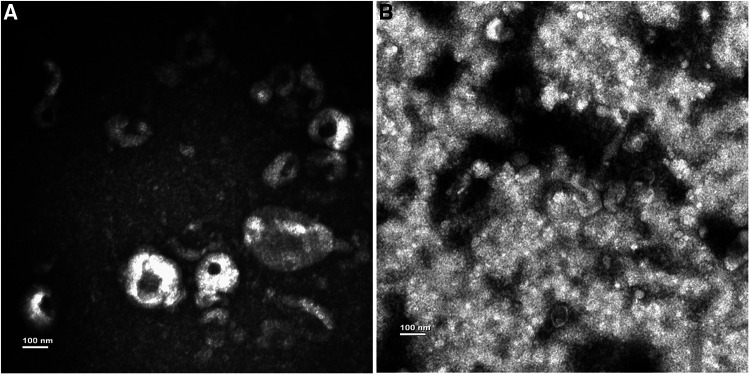
Characterization of exosomes from PE. (**A**) The exosomes from MPE. (**B**) The exosomes from TPE.

Exosomes were successfully enriched from each pleural fluid sample using the ultracentrifugation technique. Morphology of the exosome was confirmed by TEM. Representative TEM images are presented in Figure 1. TEM demonstrated that purified exosomes were round or oval with a diameter approaching 100 nm.

### MiRNAs profiling and identification of DEmiRNAs

**Figure 2:**
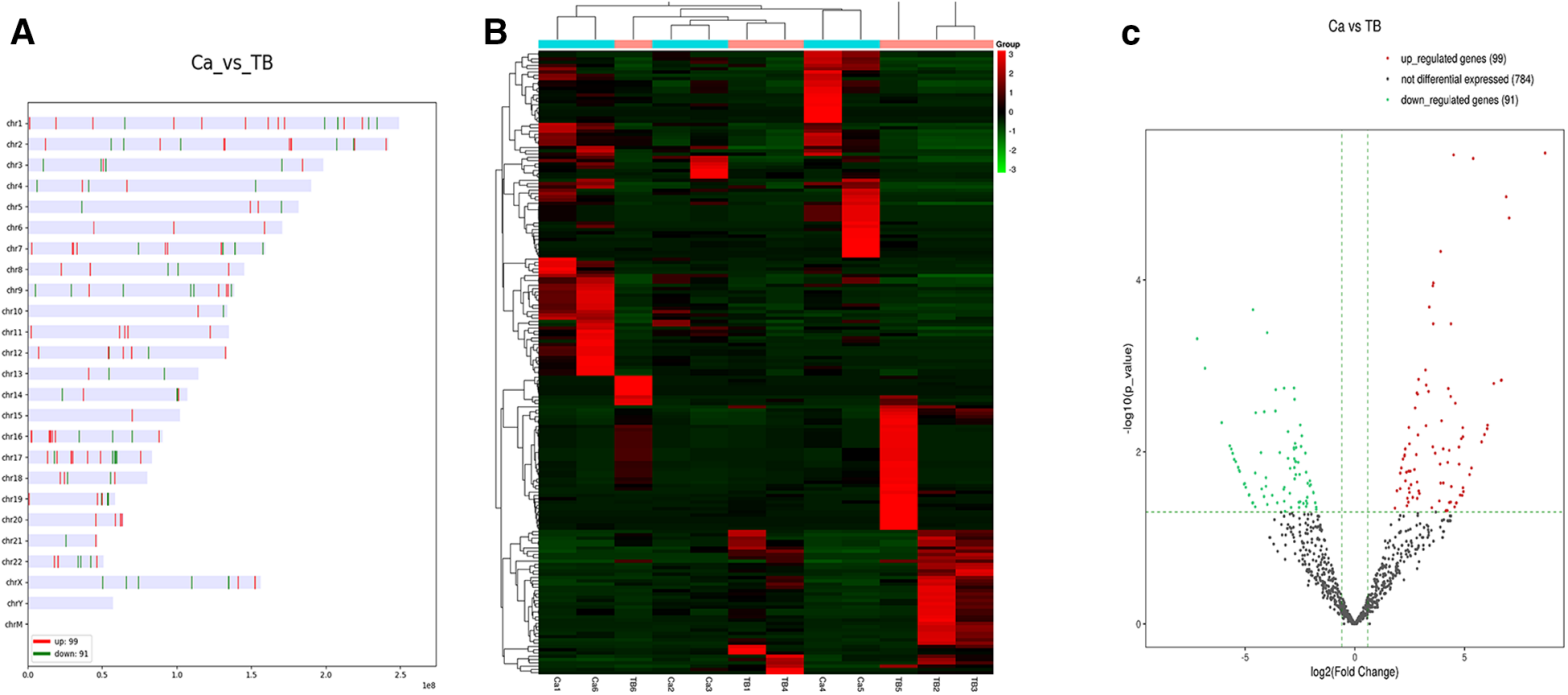
The distribution, heatmap, and volcano plot of DEmiRNAs. (**A**) The distribution of DEmiRNAs. (**B**) Heatmap of DEmiRNAs. (**C**) Volcano plot of DEmiRNAs.

This study identified 974 miRNAs in clinical samples. The DEmiRNAs were filtered based on *P *≤ 0.05 and |log2FC| ≥ 1.5. We identified 99 upregulated and 91 downregulated miRNAs in MPE compared to TPE. The top 10 upregulated and downregulated miRNAs are listed in Table 1. The distribution and heatmap of cluster analysis and the volcano plot of DEmiRNAs are displayed in Figure 2.

**Table 1:** The top 10 DEmiRNAs in exosome of MPE compared to TPE.

miRNA ID	log2FoldChange	*P*. Value
***Upregulated miRNAs***		
hsa-miR-4669-5p	8.67	3.34E-06
hsa-miR-3180-3p	7.02	1.90E-05
hsa-miR-3180-5p	7.02	1.90E-05
hsa-miR-1269b-5p	6.89	1.08E-05
hsa-miR-6884-5p	6.68	0.001462492
hsa-miR-4732-3p	6.66	0.001470397
hsa-miR-483-5p	6.33	0.00160196
hsa-miR-3120-5p	6.04	0.004874902
hsa-miR-6736-5p	6.03	0.005360191
hsa-miR-489-3p	5.90	0.006278285
***Downregulated miRNAs***		
hsa-miR-373-5p	−7.17	0.000482557
hsa-miR-519b-3p	−6.8	0.001065316
hsa-miR-4674	−6.06	0.004564553
hsa-miR-345-3p	−5.69	0.00850884
hsa-miR-4671-5p	−5.63	0.009249405
hsa-miR-7705	−5.57	0.01039574
hsa-miR-153-3p	−5.49	0.012249534
hsa-miR-33a-3p	−5.43	0.012950696
hsa-miR-4772-5p	−5.32	0.015113542
hsa-miR-580-3p	−5.27	0.016367036

### Experimental validation by qRT-PCR

**Figure 3:**
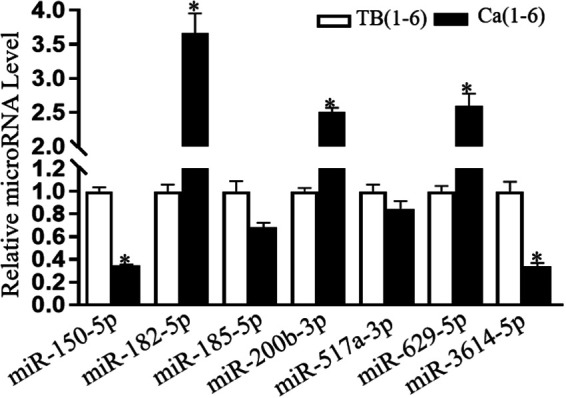
Qrt–PCR validation of miRNA sequencing results.

We randomly chose seven DEmiRNAs for qRT-PCR analysis (Figure 3). The hsa-miR-93-5p was selected as the internal reference. The results from real-time PCR analysis revealed that five of seven (71.4%) DEmiRNAs were consistent with sequencing results. Two miRNAs (hsa-miR-150-5p and hsa-miR-3614-5p) were downregulated in MPE compared to TPE. According to sequencing and PCR, hsa-miR-150-5p was down-regulated 0.18- and 0.35-fold in MPE than TPE. Three miRNAs (hsa-miR-200b-3p, hsa-miR-182-5p, and hsa-miR-629-5p) were upregulated in MPE compared to TPE. For instance, hsa-miR-629-5p was upregulated 2.61- and 3.78-fold in MPE compared to TPE, as confirmed by sequencing and PCR. However, the PCR result of hsa-miR-517a-3p and hsa-miR-185-5p were inconsistent with sequencing.

### Prediction of target genes of DEmiRNAs

To elucidate the exo-miRNAs regulatory mechanism in MPE, we predicted the target genes for DEmiRNAs using miRDB and TargetScan databases. The genes at the intersection of two gene sets were considered miRNA target genes. As a result, 305 and 739 target genes were predicted for upregulated and downregulated DEmiRNAs, respectively (Supplementary Material S1). Finally, the overlapped genes were included for further study.

### GO and KEGG analysis

**Figure 4:**
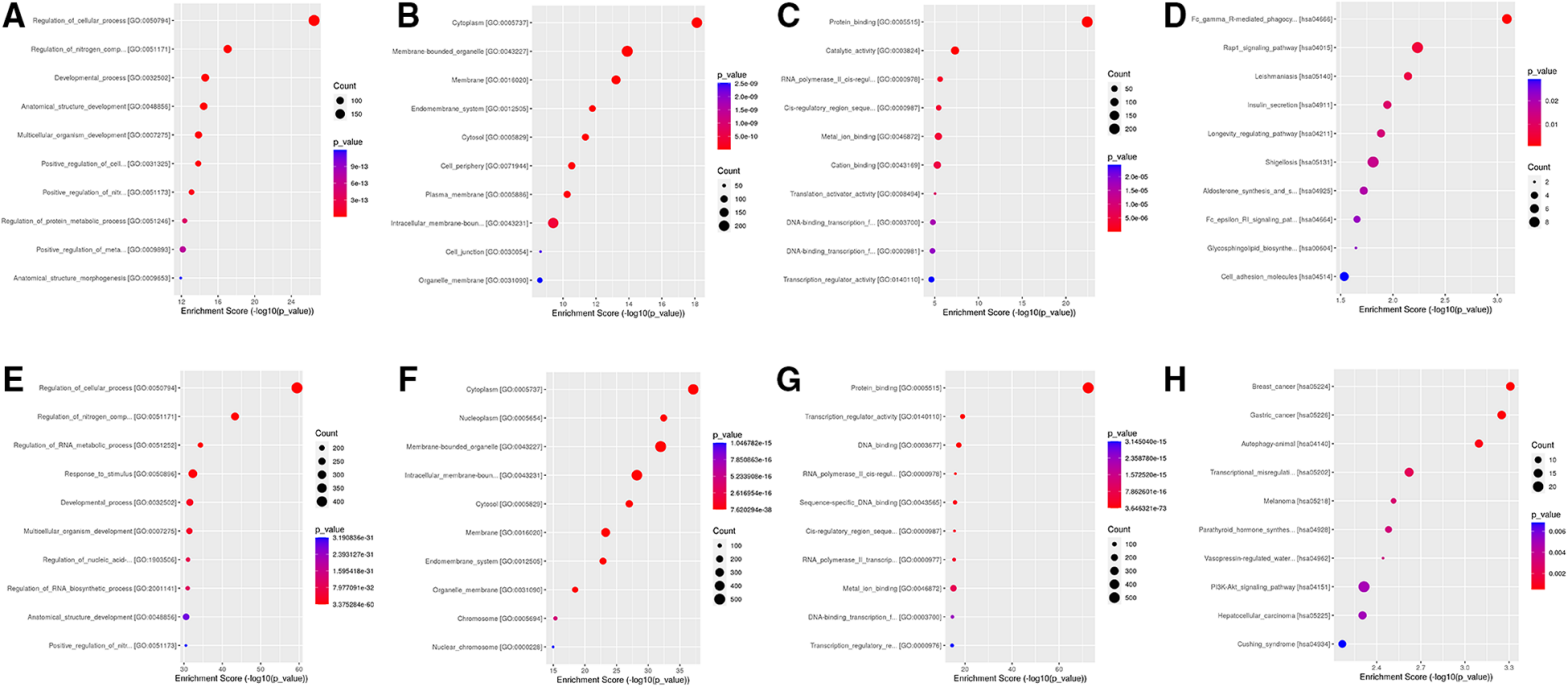
Go and KEGG analysis for target genes of DEmiRNAs. (**A**) BP group for upregulated DEmiRNAs. (**B**) CC group for upregulated DEmiRNAs. (**C**) MF group for upregulated DEmiRNAs. (**D**) KEGG for upregulated DEmiRNAs. (**E**) BP group downregulated DEmiRNAs. (**F**) CC group for downregulated DEmiRNAs. (**G**) MF group for downregulated DEmiRNAs. (**H**) KEGG for downregulated DEmiRNAs.

GO and KEGG pathway enrichment analyses were performed for the overlapped target genes**.** The top three GO analysis terms for the target genes of upregulated and downregulated DEmiRNAs are listed in Table 2. For the target genes of upregulated DEmiRNAs, the most enriched GO terms were regulation of the cellular process, nitrogen compound metabolic process, and the developmental process in the biological process (BP) group. The most meaningful GO terms were cytoplasm, membrane-bounded organelle, and membrane in the cellular components (CC) group. The most important GO terms were protein binding, catalytic activity, and RNA polymerase II cis-regulatory region sequence-specific DNA binding in the molecular function (MF) group. For the target genes of downregulated DEmiRNAs, the most significant GO terms were regulation of the cellular process, nitrogen compound metabolic process, and RNA metabolic process in the BP group. The most enriched GO terms were cytoplasm, nucleoplasm, and membrane-bounded organelle in the CC group. The most significantly enriched GO terms were protein binding, transcription regulator activity, and DNA binding. KEGG pathway enrichment analysis revealed that the target genes of upregulated DEmiRNAs were significantly enriched in Fc gamma R-mediated phagocytosis, Rap1 signaling pathway, and Leishmaniasis pathways. While the majority of the target genes of downregulated DEmiRNAs were associated with breast cancer, gastric cancer, and autophagy-animal pathways. The top ten GO and KEGG terms of the overlapped target genes are displayed in Figure 4.

**Table 2:** Go enrichment analysis result of target genes.

ID	Term	Count	*P*_value	FDR
	***Upregulated miRNA Target Genes Biological Process***			
GO:0050794	Regulation of cellular process	194	3.28e–27	1.05e–23
GO:0051171	Regulation of nitrogen compound metabolic process	116	9.00e–18	1.44e–14
GO:0032502	Developmental process	107	2.50e–15	2.68e–12
	***Upregulated miRNA Target Genes Cellular Component***			
GO:0005737	Cytoplasm	201	7.52e–19	3.20e–16
GO:0043227	Membrane bounded organelle	221	1.26e–14	2.68e–12
GO:0016020	Membrane	146	6.01e–14	8.54e–12
	***Upregulated miRNA Target Genes Molecular Function***			
GO:0005515	Protein binding	223	3.63e–23	2.37e–20
GO:0003824	Catalytic activity	99	4.74e–08	1.55e–05
GO:0000978	RNA polymerase II cis-regulatory region sequence-Specific DNA binding	31	2.47e–06	5.05e–04
* *	***Downregulated miRNA Target Genes Biological Process***			
GO:0050794	Regulation of cellular process	442	3.38e–60	1.65e–56
GO:0051171	Regulation of nitrogen compound metabolic process	275	4.98e–44	1.22e–40
GO:0051252	Regulation of RNA metabolic process	195	4.97e–35	8.08e–32
	***Downregulated miRNA Target Genes Cellular Component***			
GO:0005737	Cytoplasm	454	7.62e–38	5.06e–35
GO:0005654	Nucleoplasm	197	3.59e–33	1.19e–30
GO:0043227	Membrane-bounded organelle	510	1.08e–32	2.39e–30
	***Downregulated miRNA Target Genes Molecular Function***			
GO:0005515	Protein binding	544	3.65e–73	3.64e–70
GO:0140110	Transcription regulator activity	114	1.13e–19	5.63e–17
GO:0003677	DNA binding	129	4.20e–18	1.40e–15

### PPI network and modules analysis

**Figure 5:**
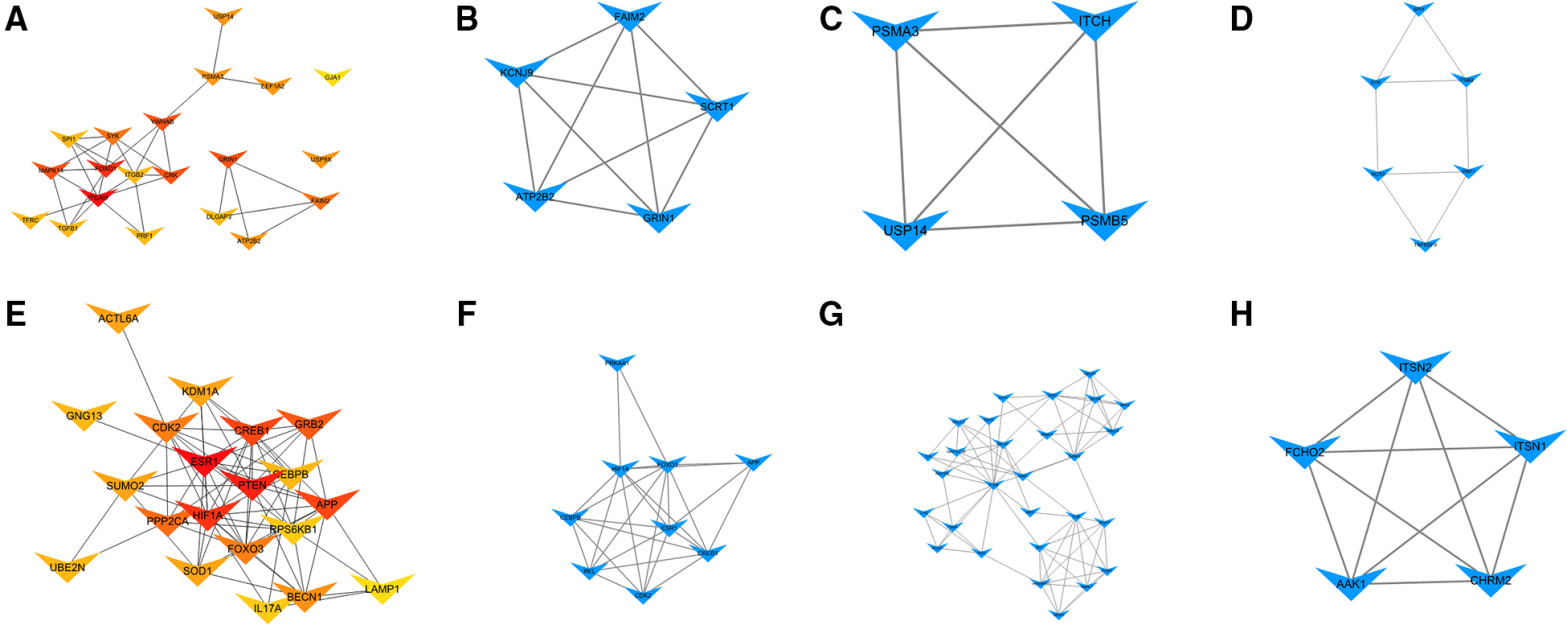
Hub genes and modules analysis. (**A**) PPI for the top 20 ranked hub genes for upregulated DEmiRNAs. (**B**) Module 1, (**C**) Module 2, and (**D**) Module 3 for target genes of upregulated DEmiRNAs. (**E**) PPI for the top 20 ranked hub genes for downregulated DEmiRNAs. (**F**) Module 1, (**G**) Module 2, and (**H**) Module 3 for target genes of downregulated DEmiRNAs.

The PPI network of target genes for DEmiRNAs was constructed by the STRING online database. Subsequently, the PPI network was visualized by cytoscape software, and hub genes were screened by degree methods through plug-in cytoHubba. The top 20 ranked genes are listed in Table 3. Three significant modules for target genes of upregulated and downregulated miRNAs were obtained using MCODE (Figure 5).

**Table 3:** the top 20 ranked hub genes.

Gene sets	Target genes of upregulated miRNAs	Target genes of downregulated miRNAs
	Gene symbol	degree	Gene symbol	degree
Hub genes	ITGAM	13	ESR1	58
	FOXO1	10	PTEN	56
	MAPK14	9	HIF1A	40
	YWHAB	9	APP	36
	GRIN1	9	CREB1	36
	CRK	9	GRB2	30
	FAIM2	8	PPP2CA	29
	SYK	8	CDK2	28
	EEF1A2	7	FOXO3	28
	USP14	7	BECN1	24
	USP9X	7	ACTL6A	22
	ATP2B2	7	SOD1	22
	PSMA3	7	KDM1A	22
	SPI1	6	SUMO2	22
	TFRC	6	GNG13	21
	PRF1	6	UBE2N	21
	TGFB1	6	CEBPB	21
	DLGAP3	6	RPS6KB1	20
	ITGB2	6	IL17A	20
	GJA1	5	LAMP1	19

### Identification of differentially expressed genes of immune cells in MPE

**Figure 6:**
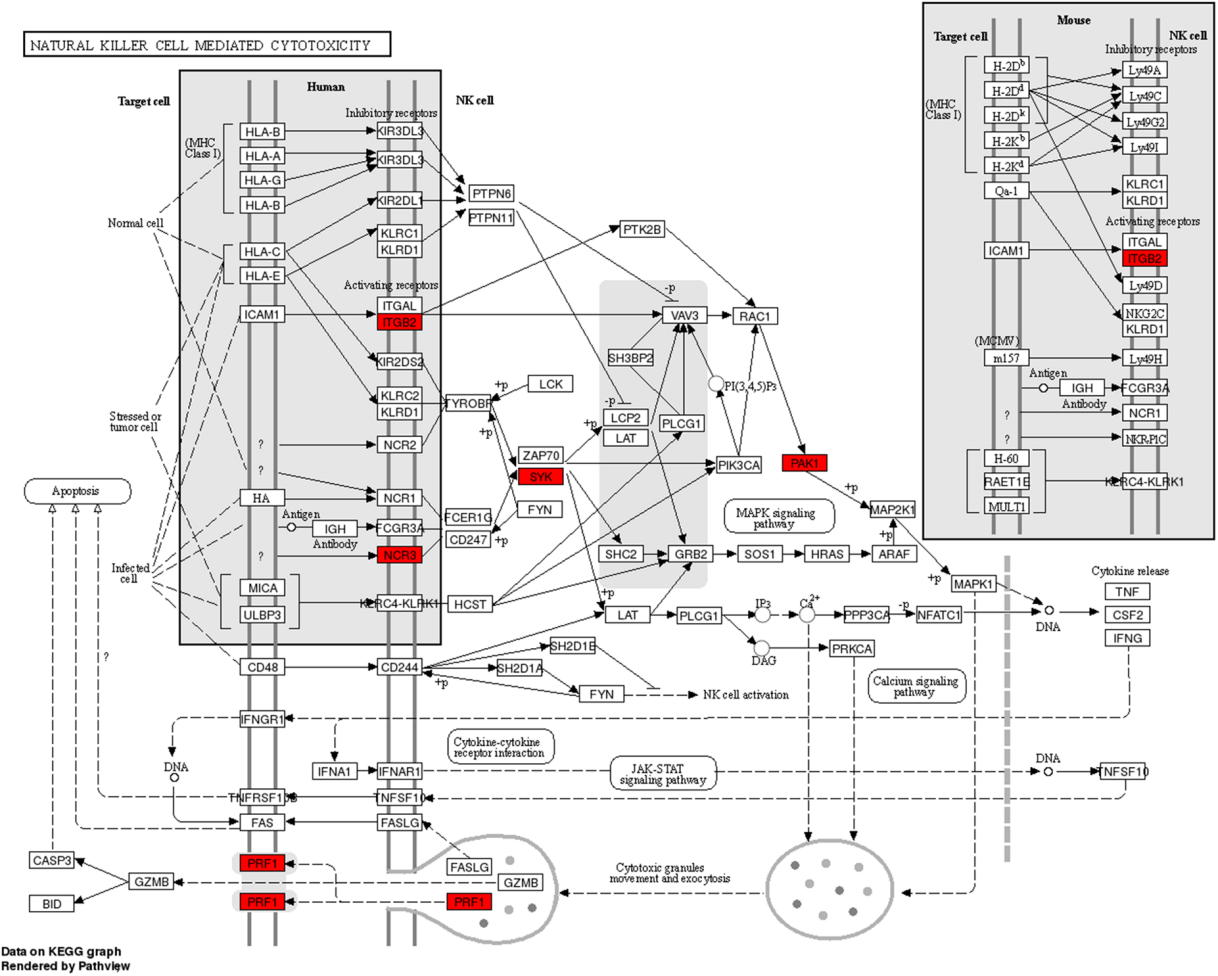
The natural killer cell-mediated cytotoxicity pathway.

Immune cells play an essential role in the pathogenesis of PE (29). Single-cell RNA sequencing was performed to explore the immunological microenvironment of MPE in GSE185058. We screened 12 overexpressed and 26 underexpressed genes (Supplementary Material S2). We crossed between 26 underexpressed genes and target genes of upregulated miRNAs and acquired a key gene, PRF1. The KEGG analysis indicated that PRF1 was involved in the natural killer cell-mediated cytotoxicity pathway (Figure 6).

## Discussion

PE is a common and devastating complication of advanced lung cancer and pulmonary tuberculosis. It is difficult to differentiate clinically between lung cancer and tuberculosis based on the pleural fluid. However, there is inconsistency in the pathogenesis of PEs due to lung cancer and pulmonary tuberculosis. In this study, we identified exosome-derived miRNA profiles and explored the underlying mechanism of PE in LUAD.

Our study demonstrated that a substantial number of miRNAs differ between MPE and TPE, especially some miRNAs that were extraordinarily highly expressed in MPE. The PCR and sequencing results confirmed that hsa-miR-200b-3p, hsa-miR-182-5p, and hsa-miR-629-5p were upregulated in MPE compared to TPE. Several studies indicated that hsa-miR-200b-3p is upregulated in renal cell carcinoma (30), gastric adenocarcinoma (31), and LUAD (32, 33). Our findings showed that hsa-miR-200b-3p was overexpressed in MPE, suggesting that it has the potential to serve as a biomarker to distinguish between benign and malignant PE. It was reported that hsa-miR-182-5p was upregulated in small cell lung cancer (34), ovarian cancer (35), and LUAD (36). Moreover, hsa-miR-629-5p was highly expressed in bladder urothelial carcinoma (37). These investigations validated the accuracy of our analysis, which implied that these miRNAs were associated with the malignant phenotypes and may aid in distinguishing between malignant and benign PE.

According to KEGG analysis, the target genes of upregulated DEmiRNAs were significantly enriched in Fc gamma R-mediated phagocytosis and Rap1 signaling pathway. Fc gamma receptors are involved in several immune system functions, such as phagocytosis and the release of inflammatory mediators. Several immune cells, including macrophages, neutrophils, and monocytes, participate in Fc gamma R-mediated phagocytosis. Many studies have demonstrated that tumor-associated macrophages and neutrophils have a pro-tumor effect, enhancing tumor cell invasion, metastasis, angiogenesis, and extracellular matrix remodeling (38). A previous study found that HUVEC cultured with malignant-associated PE promoted cell proliferation, migration, and angiogenesis (39); however, the molecular mechanism was not discussed. We speculated that the exosomes in MPE interacted with HUVEC to increase the formation of pleural capillaries and PE.

In the present study, we identified hub genes by PPI network construction. The hub gene with the highest degree for elevated miRNAs was ITGAM, which encodes for CD11b. As an immune-related gene, it regulates macrophage and monocyte activities and predicts prognosis and outcome in malignant tumors (40). It also plays an essential role in the adhesion of macrophages, neutrophils, and monocytes (41, 42). A previous study indicated that macrophages were associated with MPE and pleural metastasis of cancer triggered immune responses (43). We hypothesized that ITGAM was implicated in the development of MPE by tumor immune response, even though its significance in the PE of lung cancer is yet unknown.

Different categories of immune cells play distinct roles in the development of MPE. The wide geography of immune cells in human MPE was revealed by single-cell RNA sequencing, which also improved knowledge of the pathophysiology of MPE. In this study, we explored the DEGs of immune cells in the MPE microenvironment by analyzing single-cell sequencing data, which revealed that PRF1 was downregulated in MPE and was also identified as a hub gene. The KEGG analysis indicated that PRF1 is involved in the natural killer cell-mediated cytotoxicity pathway. The role of PRF1 in PE remains unclear. According to reports, T-lymphocyte cytotoxic function depends on the presence of PRF1, and its deficiency may impair antitumor immunity (44, 45). In this study, PRF1 was identified as the target gene of hsa-miR-3120. Hence, we proposed that the overexpression of hsa-miR-3120-5p in MPE decreases PRF1 expression and inhibits the immune response against cancer cells, leading to the formation of MPE. But there is still a lot to learn about this theory.Bioinformatic analysis depicted that the target genes of exosomal miRNAs were mainly immune-related, indicating that the tumor immune response might play a pivotal role in the generation of MPE in LUAD. Additionally, we speculated that tumor cells affected NK cells and promoted the generation of MPE in LUAD *via* the exosomal hsa-miR-3120-5p-PRF1 axis. Our results provided a new theoretical basis regarding the function of exosomal miRNAs in LUAD-MPE. However, further validation and investigation of the underlying mechanisms are required to validate our findings.

## Data Availability

The original contributions presented in the study are included in the article/**Supplementary Material**, further inquiries can be directed to the corresponding author/s.
